# Perspectives from the UK: Children and agricultural health and safety

**DOI:** 10.3389/fpubh.2022.1049150

**Published:** 2022-11-03

**Authors:** Sally Shortall

**Affiliations:** Duke of Northumberland Chair of Rural Economy, Centre for Rural Economy, School of Agriculture, Food and Rural Development, Newcastle University, Newcastle upon Tyne, United Kingdom

**Keywords:** children, farm accidents, farm family culture, lax regulation, socialization to take risk

## Current situation

The United Kingdom of Great Britain and Northern Ireland (UK) is comprised of four devolved nations; England, Scotland, Northern Ireland and Wales. The regions have very different structures of agriculture. England has some of the largest farms in Europe, with an average size of 444 hectares and many holdings in excess of 20,000 hectares. By contrast Northern Ireland has some of the smallest farms in Western Europe with an average size of 32.4 hectares (1, 2). Common across the UK is that the majority of farms are family farms. While the extent of family labor on the farm is not statistically documented, quantitative research has consistently shown that family members, including children, are very involved and often the farm relies on this unpaid labor for its viability (3–5). Average figures for the UK obscure significant differences across the regions. Yellow Wellies, a UK based charity focused on farm safety, also known as the Farm Safety Foundation, note that farming is the most dangerous occupation in the UK. In Great Britain (England, Scotland and Wales) agriculture accounts for 1% of the working population but 18% of workplace fatalities. In Northern Ireland, the figures are 3.5% of the working population and 33% of workplace fatalities (www.yellowwellies.org). The Health and Safety Executive (HSE) in Great Britain gathers data on farm fatalities and the same function is carried out in Northern Ireland by the Health and Safety Executive Northern Ireland (HSENI). In Northern Ireland the HSENI recently brought together historical injury files and made 50 years of data available to researchers in the Agri-Food and Biosciences Institute and that data has been analyzed (A2, 4). This data is not available to the public. A recent Chief Executive of the HSENI was previously at the Department of Agriculture, Environment and Rural Affairs (DAERA) and brought an understanding of farm safety to the organization. There is a comprehensive farm safety partnership in Northern Ireland that includes the regional HSENI, DAERA, Ulster farmers unions and the Farm Safety Foundation (Yellow Wellies). They run a specific “be aware kids- child safety on farms” campaign. Their activities include a primary school (children under 12) farm safety campaign and an annual calendar competition related to farm safety. The Northern Ireland Farm Safety Partnership gathered data on fatalities for 2015 and 2019. This data is from periodical reports. The HSE in Great Britain has responsibility for promoting farm safety, including that of children. In Northern Ireland, while the statistical data is less available, safety measures seem better developed. The HSE data solely documents fatalities but not wounds and life changing injuries. Yellow Wellies have tried to fill this information gap.

For the purposes of this article children are defined as 0–18 years old. In Great Britain the Farm Safety Foundation run farm safety weeks with the HSE and highlight particular dangers to children and young people. The HSE Health and Safety toolbox (103 pages) has one mention of agriculture and this relates to infection and zoonoses. More recently it has produced a specific farm safety guide (56 pages) (6). In Great Britain, each farm fatality is described in a short paragraph.

## Current challenges

The most significant challenge to improving health and safety on farms for young people is the peculiar nature of the farm. Children live and play at the worksite (7) and are immersed in the culture of farming from a young age. It is normal for children to play in the farmyard and to climb onto farm equipment. Another challenge is that despite awareness raising campaigns and safety manuals, people on farms are unaware or ignore the regulations around farming practice. A study in Scotland found that people interviewed did not know the legal age at which young people can operate machinery (5). A review of recent deaths recorded by the HSE details fatalities of children that were the result of negligent or illegal behavior. For example, in 2018/19 a 3 year old died after falling out of a farm vehicle and being crushed. It is illegal to have a 3 year old on a farm machine. A similar case of a 4 year old died in 2019/2020. In 2020/21 a 13 year old girl died when a quadbike overturned, she was not wearing a helmet contrary to HSE advice. There are many instances of such fatalities which happen annually. There is also a common myth within farming families that their children have a heightened sense of farm safety. This is despite the fact that almost all children killed on farms are farm family children. Research in Scotland found that women had this view that farm children have an innate sense of danger and understand how to be safe on farms. One woman interviewed said “*my son (three years old) wouldn't think about going out and running in front of a tractor or anything like that. Whereas he could have friends that come in that do silly things*.” Farm families have a misguided idea that their children are born understanding the dangers of the farm despite evidence to the contrary (8).

One of the biggest cultural patterns to overcome is that farm family members normalize danger and socialize each other to take risks (9, 10). Children are socialized to undertake risky behavior. Danger is normalized within farming families. Parents are aware of the dangers of the farmyard. One mother interviewed by Zepeda and Kim in the United States said that “*I always pray every day that they make it to 18 years of age*” (2006: 116). No other occupation poses this level of risk to children. Research suggests that sometimes children and young people grow up in the environment and are blind to the risks of the farm and sometimes unsafe practices are passed on from parents to children (11). In the Scottish research, a woman recounted how her 3-year-old son rides on the tractor with his grandfather, but when they attended the main national agricultural show, he could not get on the equipment. Laughingly and deridingly she recounted; “*He couldn't understand why at the Highland Show*^1^ *he couldn't get on the tractor to drive it! Because it's locked son! You can't get on! You can't get on it! Health and safety son!”*

Research in northern Ireland found that women who worked off farm worried about children coming home to empty houses, and they worried about children having to walk on dark country roads because school buses drop children a long way from the farm (4). One mother said “*there are more checks on calves than children*.” Research in Scotland (5) also found a mother who worried about leaving her small children in the house when doing farm work. Her partner worked off farm and she felt she had no other option but to leave the children alone.

## Future directions

Currently the farmyard, despite being the most dangerous workplace, is very lightly regulated in the UK. Planning regulations and health and safety regulations make exemptions for agriculture that they do not for any other occupation, allowing it to persist as a dangerous occupation. In particular exemptions are made which allow younger children to drive farm machinery than is the norm for non-farm children. While young people cannot drive cars until they are seventeen years old, exceptions are made for farm children who are allowed to drive heavy machinery at thirteen and fourteen years of age as long as it is on the farm property, where the majority of injurie occur. The heredity nature of farming and the involvement of the family from a young age suggests that it is seen as a special, pre-industrial occupation to which rules do not apply as elsewhere. Hard questions need to be asked about why this hazardous occupation has so many exemptions from planning regulations and health and safety regulations.

The sanctions for failing to adhere to safe practice are light. The current loose regulatory framework around farm safety practice contributes to the view that farm danger is normal and what happens on the farm is beyond scrutiny. The UK does not need more information about the hazards faced by children on farms rather the challenge is changing farm family culture. Families ignore, or are unaware of, legal requirements. Health and Safety Executives, farm families and farming bodies must work closer together to ensure farms become safer places. Farm safety partnerships need to consider the type of support needed by farm families, such as where children are dropped by school buses and what type of childcare provision could be tailored for farm families.

## Author contributions

The author confirms being the sole contributor of this work and has approved it for publication.

## Conflict of interest

The author declares that the research was conducted in the absence of any commercial or financial relationships that could be construed as a potential conflict of interest.

## Publisher's note

All claims expressed in this article are solely those of the authors and do not necessarily represent those of their affiliated organizations, or those of the publisher, the editors and the reviewers. Any product that may be evaluated in this article, or claim that may be made by its manufacturer, is not guaranteed or endorsed by the publisher.

## Footnotes

^1^A large Scottish agricultural show.
